# Patients’ Perceptions and Satisfaction with the Outpatient Telemedicine Clinics during COVID-19 Era in Saudi Arabia: A Cross-Sectional Study

**DOI:** 10.3390/healthcare9121739

**Published:** 2021-12-16

**Authors:** Ashokkumar Thirunavukkarasu, Nasser Hanas Alotaibi, Ahmad Homoud Al-Hazmi, Mohammed Jayed Alenzi, Ziad Mansour Alshaalan, Mohammed Ghazi Alruwaili, Thamer Alshami Marghel Alruwaili, Hassan Alanazi, Turki Hanas Alosaimi

**Affiliations:** 1 Department of Community and Family Medicine, College of Medicine, Jouf University, Sakaka 72388, Saudi Arabia; ahhazmi@hotmail.com; 2 College of Medicine, Jouf University, Sakaka 72388, Saudi Arabia; nas.alosaimi@hotmail.com; 3 Department of Surgery, Division of Urology, College of Medicine, Jouf University, Sakaka 72388, Saudi Arabia; mja@ju.edu.sa; 4 Department of Internal Medicine, Division of Dermatology, College of Medicine, Jouf University, Sakaka 72388, Saudi Arabia; dr.ziad@ju.edu.sa; 5 Qurayyat Health Affairs, Aljouf, Ministry of Health, Qurayyat 77461, Saudi Arabia; alrwuili.m@gmail.com; 6 Department of Pediatrics, College of Medicine, Jouf University, Sakaka 72388, Saudi Arabia; tsruwaili@ju.edu.sa; 7 Department of Obstetrics and Gynecology, King Khalid University Hospital, Riyadh 12372, Saudi Arabia; hasan.alanzi@gmail.com; 8 Department of Hospital Management Services, Huraymala General Hospital, Riyadh 11911, Saudi Arabia; t.alosaimi1@gmail.com

**Keywords:** COVID-19, perceptions, satisfaction, Saudi Arabia, virtual clinics

## Abstract

Successful implementation of virtual healthcare depends immensely on patients’ perceptions and satisfaction. This cross-sectional study assessed patients’ perceptions of, and factors associated with, poor and average satisfaction with the outpatient telemedicine clinics in the Kingdom of Saudi Arabia (KSA). This questionnaire-based survey was conducted among 720 patients who attended outpatient telemedicine clinics from different regions of the KSA. Of the sample studied, 54.7% of the participants had high satisfaction and the most common disadvantage perceived by patients was technical issues (53.1%), followed by fewer personal interactions (30.4%). Around 75% of the participants desired to use telemedicine services even after the COVID-19 pandemic. Logistic regression analysis revealed that age group more than 40 years (OR = 1.59; 95% CI = 1.04–2.44, *p* = 0.031), education less than university level (OR = 1.68; 95% CI = 1.07–2.15, *p* = 0.025), and first-time participants (OR = 3.28; 95% CI = 2.32–4.65, *p* < 0.001) were significantly associated with poor and average satisfaction ratings. The concerned authorities must make targeted action plans to circumvent the disadvantages perceived by patients accessing telemedicine. Furthermore, a multicenter, exploratory study that compares the virtual clinic with other telemedicine services in the KSA is warranted.

## 1. Introduction

The World Health Organization (WHO) elucidated telemedicine as “The delivery of healthcare services, where distance is a basic factor, by all medical care experts utilizing information and communication technologies (ICTs), for diagnosis, treatment, and prevention of diseases and infirmities” [1]. Telemedicine incorporates the use of telecommunications in physician–patient interactions [2]. Telemedicine implies a medical care supplier’s utilization of ICTs in the conveyance of clinical medical care administration. In contrast, telehealth alludes to a medical care supplier’s utilization of ICTs to convey clinical, as well as non-clinical, medical services administration [2,3]. Accessibility, equity distribution, quality, and cost-effectiveness are the essential challenges that healthcare systems face worldwide. Modern ICTs, such as smartphones, the internet, and computers, have great potential to address the contemporary health problems of developed and less developed countries [1,4].

The first telemedicine programs were established almost 70 years ago, but the technology grew considerably after 2019 and has become widely interactive in all surgical and medical specialties because of the COVID-19 pandemic and lockdown [5,6,7]. One of the advantages of telemedicine is cost saving because transmitting information is less expensive than transporting people. Other benefits include immediate access to medical expertise regardless of location, more timely diagnoses and treatments than might be possible, and the elimination of long patient commutes from rural communities to urban centers [8,9,10]. Telemedicine has been extensively utilized during the COVID-19 pandemic as it helps in diminishing contact with medical services offices, staff, and patients, and thereby minimizes the danger of COVID-19 spread [11].

Successful implementation of any healthcare delivery, including telemedicine, depends immensely on patient perceptions and satisfaction. Patients are the primary source of information that tells us whether the healthcare is being delivered properly and if the healthcare received meets their expectations [12,13,14]. Dissatisfaction with telemedicine care services would render these services unnecessary and costly. With the surge in worldwide telemedicine services during the COVID-19 pandemic, it is essential to maintain a key quality evaluation indicator of patient satisfaction, regardless of delivery method [15]. Patient satisfaction is the voice of the customer in healthcare and is a growing concern in all aspects of healthcare. Just like the conventional modalities of medical care conveyance, telemedicine depends intensely on feedback from patients [16,17]. A systematic review conducted by Kruse et al. revealed that assessing patients’ satisfaction toward telehealth and its associated factors could help stakeholders to find solutions for specific problems [15]. A recent study by Asma et al. in the Kingdom of Saudi Arabia (KSA) reported that nearly one-third (37%) of patients were highly satisfied with the telemedicine services provided to them during the COVID-19 pandemic [18].

In the KSA, the Ministry of Health (MOH) is concerned with telemedicine through different platforms such as outpatient telemedicine clinics (virtual clinics), 937 call centers, and the Seha smartphone application. In addition, the Saudi Commission for Health Specialties also launched a “telemedicine” training program to train all healthcare professionals to care for patients remotely with the best global practices in telemedicine [19]. Continuous assessment of patients’ perceptions and satisfaction towards telemedicine and its associated factors is essential for the successful implementation of high-quality telemedicine care, especially during pandemics. In the KSA, some authors attempted to find patient satisfaction towards telemedicine [18,20]. However, studies that investigate patients’ perceptions of its advantages and challenges, as well as factors associated with poor satisfaction towards telemedicine, are limited. Therefore, the present study aimed to assess patients’ perceptions of and satisfaction with the Outpatient Telemedicine Clinics, and the association between socio-demographic characteristics and satisfaction scores during the COVID-19 era in the KSA.

## 2. Materials and Methods

### 2.1. Study Design and Setting

This analytical, cross-sectional study was performed from 15 June 2021 to 20 October 2021. The present study’s setting was outpatient telemedicine clinics from different departments registered with the MOH, KSA.

### 2.2. Sample Size Estimation

The research team estimated the required sample size based on the formula *n* = z^2^ pq/e^2^. Here, “*n*” is the required sample size, “p” is prevalence (37.3% of patients’ satisfaction towards telemedicine) taken from the study done by Nasser et al. [18], q is 1−p (1−0.373) = 0.627, z = 1.96 at 95% confidence interval, and e = 5% margin of error. Applying the values to the above formula, the estimated minimum required sample size for this study was 360 participants. Considering the inclusion of multiple regions of the KSA, we doubled (720) the sample size for this study.

### 2.3. Sampling Procedure

A consecutive sampling method was applied to select study participants. In this method, the data collectors communicated with the consecutively registered patients after they completed their outpatient telemedicine clinic visits. To get patients from all the days of the week for the survey, we restricted contact to a maximum of 50 patients per day for data collection. 

### 2.4. Inclusion and Exclusion Criteria

The present study included patients attending all the department telemedicine outpatient virtual clinics concerned with the MOH, KSA. Patients who were not willing to participate and private hospital telemedicine clinics were excluded from the present study. Further, this study did not include patients attending other MOH telemedicine care platforms (937 call centers, the Seha app, etc.).

### 2.5. Ethical Consideration

The present study’s data were collected after ethical committee clearance (no. 089, Qurayat Health Affairs, MOH, KSA) and other necessary approvals were obtained from the concerned authorities. Informed consent was obtained from each participant before proceeding to fill the data collection form.

### 2.6. Data Collection Procedure

An open-source, structured, and validated questionnaire (Cronbach’s Alpha = 0.89) adapted from a previously published study [18] was sent to the selected patients through their WhatsApp or email immediately after telemedicine consultation, or, at the maximum, the end of that day (by Google form). The questionnaire consisted of three parts. Part 1 collected details about patient socio-demographic characteristics. Part 2 contained details related to patient perception towards telemedicine, including its perceived advantages and disadvantages. Part 3 had eight questions related to patient satisfaction related to telemedicine. The responses in each question were recorded on a 5-point Likert scale as very satisfied to very dissatisfied. The score for each response ranged from 5 (very satisfied) to 1 (very dissatisfied). At the end of the survey, the total score was calculated. A higher score in the patient satisfaction questionnaire indicated higher satisfaction with telemedicine services. Furthermore, patient satisfaction was graded as high (>75% of total score), average (50 to 75% of total score) and poor (<50% of total score).

### 2.7. Statistical Analysis

Statistical Package for Social Sciences (SPSS) software version 21 (IBM, Armonk, NY, USA) was used for data exported from Excel sheets obtained from Google form. Descriptive statistics for qualitative variables were presented as frequency and percentage, and mean and standard deviation (SD) for quantitative variables. After the Wilk–Shapiro test analysis, we executed independent t test and one way analysis of variance (ANOVA) to find the association between socio-demographic variables and mean satisfaction scores. We performed binomial logistic regression (enter method) analysis to identify the factors associated with poor and average telemedicine satisfaction. In this method, the adjusted independent variables were age category, gender, marital status, education status, employment, Virtual clinic setting, telemedicine before pandemic, and consultation department. A *p*-value of less than 0.05 and an odds ratio (OR) that did not include the null value were considered as statistically significant.

## 3. Results

Of the 720 participating patients, the majority (55%) of them were of male gender, married (65%), studied at the university/college level (82.5%), and employed in government settings (63.6%). Further, less than one-third of participants (29%) had experienced a telemedicine consultation before the COVID-19 pandemic (Table 1).

**Figure 1:**
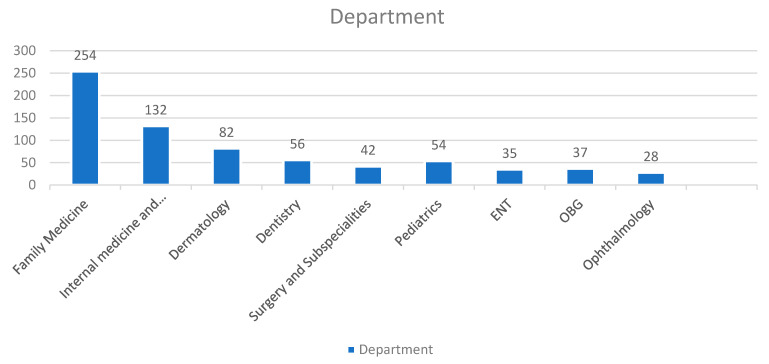
Patients’ distribution according to department.

Figure 1 demonstrates the participating patients’ distribution as per the department/specialty outpatient virtual clinics. Of the 720 patients, 254 (35.2%) attended family medicine, followed by 132 (18.3%) in internal medicine and subspecialties, 82 (11.4%) in dermatology, and 56 (7.8%) in dentistry virtual outpatient clinics.

The relationship between socio-demographic characteristics and mean satisfaction scores towards virtual clinics is presented in Table 2. The mean satisfaction scores were significantly higher among the age group less than 40 years (mean ± SD = 32.93 ± 4.4, *p* < 0.001) and the married group (mean ± SD = 32.76 ± 5.4, *p* = 0.004). No other parameters had shown a significant association.

Table 3 presents the patients’ perception of virtual clinics, perceived advantages and disadvantages, and suggestions for improving telemedicine visits. Of the 720 participants, nearly half (45.3%) of them responded that their desire to be seen in person by the healthcare provider has changed during the current pandemic. Nearly two-thirds (68.1%) of the participants were willing to participate in another telemedicine consultation during the pandemic, and 74.4% of patients preferred telemedicine consultation even after the COVID-19 pandemic. The common advantages of telemedicine perceived by patients were convenience (79.3%), time saving (62.8%), and availability of the healthcare provider (45.7%). More than half (53.1%) of the participants responded that technical difficulties was the most common disadvantage perceived by them.

**Figure 2:**
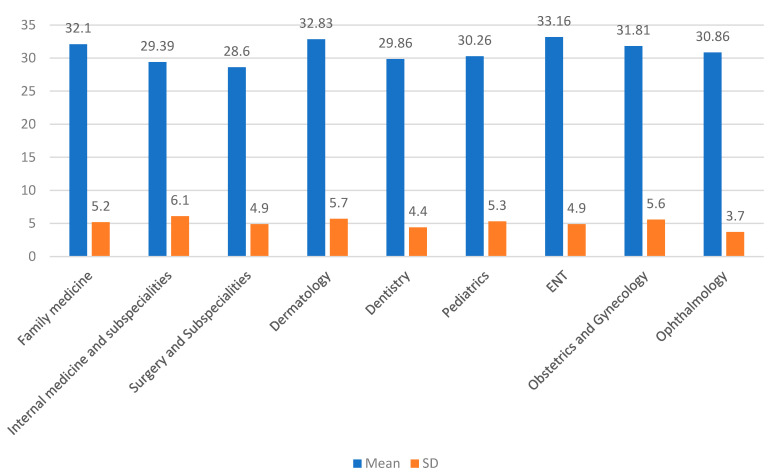
Distribution of patients’ satisfaction scores as per the specialties (mean and SD).

In the present study, the highest mean score was noted in ENT (33.16 ± 4.9), followed by dermatology (32.83 ± 5.7) and family medicine (32.1 ± 5.2) (Figure 2).

**Figure 3:**
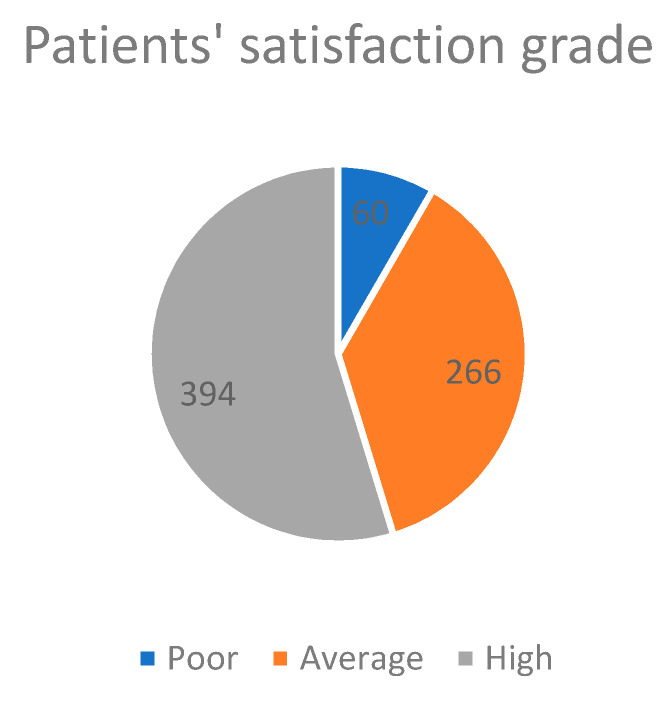
Patients’ satisfaction grade for telemedicine (*n* = 720).

Of the 720 participants, 394 (54.7%) had high satisfaction, 266 (36.9%) had average satisfaction, and 60 (8.3%) had poor satisfaction with the virtual outpatient telemedicine clinics (Figure 3).

In the present study, we performed binomial logistic regression analysis to find the associated factors for poor and average patient satisfaction towards telemedicine. The research team executed the logistic regression analysis and, adjusting with other co-variables, the characteristics that were significantly associated with poor and average patient satisfaction scores were age group more than 40 years (OR = 1.59; 95% CI = 1.04–2.44, *p* = 0.031), education less than University/College level (OR = 1.68; 95% CI = 1.07–2.15, *p* = 0.025), patients attending a specialty hospital (OR = 1.73; 95% CI = 1.21–2.47, *p* = 0.03), and patients who had never had a telemedicine experience before (OR = 3.28; 95% CI = 2.32–4.65, *p* < 0.001) (Table 4).

## 4. Discussion

Globally, healthcare delivery has gone through a paradigm shift during the COVID-19 pandemic, with a sharp increase in virtual care. The promotion of telemedicine care might play a significant role in providing and accessing virtual care and other telehealth services during public health emergencies, such as the COVID-19 pandemic, as stated by the Centers for Disease Prevention and Control [21].

The present study found that 45.3% of participants agreed that the COVID-19 pandemic had changed their desire to see healthcare providers in person. Similar to the present research, Holtz et al. and Khan et al. also found that their study participants changed their desire to see providers [22,23]. This attitude change among patients is due to fear of exposure to COVID-19 patients in healthcare settings, as the world is far from the end of the pandemic.

The present study depicted that only 29% of the participants were involved in any form of telemedicine before the COVID-19 pandemic. However, an increased number of participants (74.4%) expressed their desire to have telemedicine consultations once the pandemic is over. This interesting finding suggests that patients wish to transform their healthcare towards virtual care in necessary situations. On the other hand, a study done by Grossman Z. et al. in 2021 stated that healthcare providers envisage decreasing virtual care after the pandemic [24]. Similar to the current study findings, Lagasse J. also reported that most consumers wished to prefer telemedicine even post-COVID-19 era [25]. In contrast, Nasser A. et al. reported a lesser proportion (48.9%) of patient preference towards telemedicine once the COVID-19 pandemic is over [18]. This striking difference between the different studies could be due to the differences in study settings, inclusion criteria, and the departments involved. The present study included patients from all medical and surgical specialties from regions of the KSA.

Technical difficulties (53.1%), poor communication (34.4%), and less personal communication (30.4%) were the most common disadvantages perceived by the present study’s participants. Similarly, Nasser A. et al. and Alharbi et al. also reported that technical difficulties was the most common disadvantage perceived by patients [18,20]. On the other hand, physicians faced difficulties in making proper clinical diagnoses due to the absence of physical examinations, which was their major perceived disadvantage of virtual care [26].

The present study revealed that more than half (54.7%) of the study participants were highly satisfied with their telemedicine services. Similar to the present survey, a study done in the KSA during the COVID-19 pandemic also revealed that 52% of participants were highly satisfied with telemedicine services [18]. This level of acceptance and satisfaction with telemedicine services in the KSA could be positively influenced by internal or external circumstances. External circumstances refers to the system providing telemedicine care, including government policy towards digital services, availability of trained healthcare providers, and advanced network coverage (4G/5G). At the same time, the internal factors are socio-cultural, education, economic status, and high-level acceptance of innovation [27,28,29]. It is noteworthy to mention that the Saudi Commission for Health Specialties regularly conducts telemedicine training programs for all healthcare professionals to attain telemedicine care at a global level [19]. Other factors responsible for high-level satisfaction in the KSA are the high level of literacy (95.3%) and coverage proportion (90%) of advanced internet in the KSA. The research communities revealed the same high-level satisfaction in other economically developed countries [17,30]. However, the developing and underdeveloped countries’ scenarios could be different due to low literacy rates, poor economies, poor network coverage, unreliable electric power, and restricted resources to implement new technologies [31,32,33].This study aimed to identify the factors associated with poor and average satisfaction towards outpatient telemedicine clinics through binomial logistic regression. The present study revealed that higher age group was one of the significant variables associated with poor and average satisfaction (OR = 1.59; 95% CI = 1.04–2.44, *p* = 0.031). Similar to the present study findings, several authors also reported that increasing age is a significant factor for poor telemedicine satisfaction [20,23,34,35]. In the era of rising virtual care, aged people find several barriers when accessing it due to inadequate technical competencies and visual and hearing difficulties. This leads to poor acceptance of and dissatisfaction with telemedicine among them. It is noteworthy to mention here that the United Nations observed the international day of older people in 2021 with the theme “Digital Equity for All Ages.” This emphasizes the need for access to care and meaningful involvement in digital healthcare by aged persons [36]. In this study, participants with less than university education levels had significantly lower satisfaction with outpatient telemedicine clinics (OR = 1.68; 95% CI = 1.07–2.15, *p* = 0.025). Identical to the present research findings, Khan Z. et al. and Alharbi et al. also reported a significant association between patients’ satisfaction with virtual clinics and level of education [20,23]. This could be due to the influence of education on health and ease of using technology among educated patients [37].

The present study depicted that those patients who attended virtual clinic consultations at specialty hospitals had significantly lower satisfaction levels (OR = 1.73; 95% CI = 1.21–2.47, *p* = 0.03). In the KSA, specialty hospitals generally provide more advanced care for patients. Hence, patients might have preferred to be seen by healthcare providers in person for detailed clinical examinations and laboratory investigations. Another significant variable identified in our study that is associated with the lower satisfaction regarding the virtual clinics is first-time virtual care attendees (OR = 3.28; 95% CI = 2.32–4.65, *p* < 0.001). Since virtual healthcare delivery is a relatively new concept, patients who attended first-time virtual care might not know how to use it. This finding is supported by a study done in the primary health care settings in the Riyadh region of the KSA [20]. In that research, well-informed patients had significantly higher satisfaction with virtual clinics. Healthcare providers, including physicians, are the essential stakeholders for implementing telemedicine services. A study conducted by Alhajri et al. in 2021 at Abhudhabi reported that physicians perceived that spending less time in documentation and seeing more patients within their duty schedule were the significant advantages of telemedicine. They also perceived that telemedicine could be more effective in follow-up, reordering medications, psychological fields, and essential primary care services. However, a high chance of misdiagnosis due to lack of physical examination and less personal rapport with patients were the disadvantages perceived by the physicians. Furthermore, the physicians were more confident in delivering care with combined audio-video care than with audio care alone [38]. Another study that assessed perceived advantages and disadvantages among Saudi physicians in the Taif region showed that almost a third of the participants believed that telemedicine increased the effectiveness of therapeutic intervention and 44% perceived that virtual care improved patient care. However, the physicians reported that virtual care cannot replace the face-to-face consultation and it can be used for stable patients from remote areas [26]. Some authors explored the integrative advantages and disadvantages perceived by the patients and care providers of telemedicine. They suggested that patient satisfaction and quality of care can be improved by audio-video consultation care, limiting access to such care by stable patients, and referral care for face-to-face examinations where physicians suspect the possibility of misdiagnosis due to lack of physical examination [39,40].

Even though the research team executed this cross-sectional survey with a standard methodology, certain limitations need to be noted while reading the results of this study. Firstly, this study used a non-probability consecutive sampling method to select the study participants. Hence, limitations associated with this method are applicable to the present study. Secondly, this cross-sectional study attempted to find only association, not causation.

## 5. Conclusions

The present study revealed that more than half of the participants had high satisfaction with outpatient telemedicine clinics. This study depicted several socio-demographic factors associated with poor and average patient satisfaction. Even though the patients perceived some disadvantages, technical issues was the major disadvantage faced by them. Most of the participants desired to use telemedicine services even after the COVID-19 pandemic. This indicates that the COVID-19 pandemic has transformed KSA patients’ desire towards virtual care. However, the concerned authorities must make the necessary and targeted action plans to circumvent the disadvantages perceived by patients regarding telemedicine. Furthermore, a multicenter, exploratory study that compares the virtual outpatient clinic with other telemedicine services provided by the MOH, KSA is warranted.

## Figures and Tables

**Table 1:** Background details of the study participants (*n* = 720).

Variables	Frequency (*n*)	%
Age group (in years) (mean ± SD)	42.9 ± 9.1	
Gender Male Female	396 324	55.0 45.0
Marital status Married Single	468 252	65.0 35.0
Education level Less than University/College University/College	126 594	17.5 82.5
Employment Government Self-employed/Private Unemployed	458 120 142	63.6 16.7 19.7
Telemedicine consultation at PHC General hospital Specialty hospital	302 340 78	41.9 47.2 10.8
Telemedicine consultation experience before the pandemic Yes No	209 511	29.0 71.0

**Table 2:** Comparison of telemedicine scores with patients’ background characteristics.

Variables	Mean ± SD	*p*-Value (Two Tailed)
Age (in years) * ≤40 years >40 years	32.93 ± 4.4 30.31 ± 4.7	<0.01
Gender * Male Female	31.41 ± 4.9 31.23 ± 3.8	0.691
Marital status * Married Single	32.76 ± 5.4 30.59 ± 6.4	0.004
Education * University/College Less than University/College	31.79 ± 4.1 31.26 ± 5.9	0.374
Employment status ** Government Self-employed/Private Unemployed	31.43 ± 4.3 32.02 ± 3.8 31.75 ± 5.1	0.652
Telemedicine consultation at ** PHC General hospital Specialty hospital	32.46 ± 5.7 32.74 ± 4.9 30.18 ± 5.1	0.07
Telemedicine consultation experience before the pandemic * Yes No	31.23 ± 3.4 32.14 ± 3.7	0.105

* Independent t test, ** One way ANOVA.

**Table 3:** Patients’ perceptions towards virtual outpatient clinic consultation (*n* = 720).

Perceptions	Frequency (*n*)	%
Has the COVID-19 pandemic changed your desire to be seen in person by a healthcare provider? Yes No Not sure	326 186 208	45.3 25.8 28.9
Do you think anything was missed or not addressed because you were not seen in person? Yes No Not sure	284 214 222	39.4 29.7 30.8
Willingness to participate in another telemedicine consultation during the COVID-19 pandemic Yes No Not sure	490 124 106	68.1 17.2 14.7
Preference towards telemedicine consultation once the COVID-19 pandemic is over. Yes No Not sure	536 126 58	74.4 17.5 8.1
Perceived advantages towards telemedicine Availability of healthcare provider Convenience No time off from work No travel Time saving Safety Visits not rushed None	329 571 289 128 452 96 85 53	45.7 79.3 40.1 17.8 62.8 13.3 11.8 7.4
Perceived disadvantages towards telemedicine Technological difficulties Less personal interaction Poor communication None	382 219 248 110	53.1 30.4 34.4 15.3
Recommendation for telemedicine care improvement Improvement in scheduling/coordination Improved technology Incorporation of diagnostic recommendation None	281 320 162 91	39.0 44.4 22.5 12.6

**Table 4:** Logistic regression analysis on patients’ socio-demographic characteristics with poor and average satisfaction towards telemedicine.

Characteristics	Total Sample (*n* = 720)	Poor and Average Satisfaction		Multivariate Analysis * No vs. Yes	*p* Value **
		No (*n* = 394) *n* (%)	Yes (*n* = 326) *n* (%)	Adjusted OR (95% CI)	
Age (in years) ≤40 years >40 years	286 434	190 204	96 230	Ref 1.59 (1.04–2.44)	0.031
Gender Female Male	324 396	174 220	150 176	Ref 0.55 (0.89–0.63)	0.554
Marital status Single Married	252 468	166 228	86 240	Ref 1.51 (0.96–1.91)	0.072
Education University/College Less than University/College	594 126	326 68	268 58	Ref 1.68 (1.07–2.15)	0.025
Employment status Government Self-employed/Private Unemployed	458 120 142	226 72 96	232 48 46	Ref 1.38 (0.81–2.35) 0.98 (0.56–1.74)	0.237 0.932
Telemedicine consultation at PHC General hospital Specialty hospital	302 340 78	203 167 24	99 173 54	Ref 0.57 (0.31–1.05) 1.73 (1.21–2.47)	0.70 0.03
Previous telemedicine consultation Yes No	209 511	130 264	79 247	Ref 3.28 (2.32–4.65)	<0.001
Consultation department Family medicine Internal medicine and subspecialities General Surgery and subspecialties Dentistry Other departments	254 132 42 56 236	156 62 26 18 132	98 70 16 38 104	Ref 1.52 (0.95–2.43) 1.08 (0.51–2.28) 1.31 (0.92–2.13) 1.48 (0.98–2.24)	0.081 0.844 0.142 0.061

* Variable(s) entered on step 1: age category, gender, marital status, education status, employment, virtual clinic setting, telemedicine before pandemic, and consultation department. ** *p* value less than 0.05 was considered as statistically significant.

## Data Availability

The data presented in this study are available on request from the corresponding author.
